# Factors explaining the dominion status of female sterilization in India over the past two decades (1992-2016): A multilevel study

**DOI:** 10.1371/journal.pone.0246530

**Published:** 2021-03-10

**Authors:** Pragya Singh, Kaushalendra Kumar Singh, Pooja Singh

**Affiliations:** Department of Statistics, Institute of Science, Banaras Hindu University, Varanasi, Uttar Pradesh, India; Institute of Economic Growth, INDIA

## Abstract

**Background:**

Female sterilization is a permanent method of contraception practiced widely in India. Though, the important evidences of behavior of contraceptives is widespread in the literature, relatively less research has been conducted that explores particularly female sterilization method and how its behavior has remained dominant over the past two decades. The present study aims to examine how the level of women’s socio-demographic and fertility related characteristics intersect to shape the behavior for the dominance of female sterilization.

**Methods:**

This study was based on pooled data from 1992–93, 1998–99, 2005–06 and 2015–16 India’s DHS (NFHS) surveys. The outcome variable of the study was different types of contraceptive methods used. Multinomial logistic model has been applied to examine the relationship between the dependent variable and the explanatory variables. The software STATA version14 has been used for the entire analysis.

**Result:**

The result of this study clearly demonstrates the evidence of continuing sterilization dominance in the India’s family planning program. The choice of different types of contraceptive methods is influenced by the longstanding heterogeneity of population associated with religion and the caste system. Reliance over female sterilization was observed in almost all parts of the country with southern India being the leading zone. Women in the lowest wealth quintile, uneducated, higher parity, and less exposed to media were more likely to use sterilization as a method of birth control.

**Conclusion:**

The study was successful in identifying the factors behind the excessive dependency on female sterilization and also highlights the weakness of family planning program to promote other useful modern methods over the past two decades.

## Introduction

Overpopulation has been a major concern of India since independence. In 1952, India became the trailblazer in the world to launch a nationwide family planning program with the primary intention to control its rapidly increasing population [1–3]. In spite of being the world’s first country to launch the family planning program and devote a particular department for this purpose named as the department of Health and Family Welfare, India is one the country where most sterilization are performed in the world, in terms of both absolute numbers and percentage of the population [4]. Though history of family planning holds the evidences of the approach behind it being controversial, but significant achievements have been noticed in the recent decades in this regard. In the early phase of program natural method particularly rhythm was promoted by several demographers and policy makers [5]. But due to inconsistent use of this method and the occurrence of major breakthrough in technologies, the government started recommending modern methods such as condom, jelly, diaphragms over traditional methods [6].

Later in 1966, method acceptance shifted towards the permanent method, mainly centered on male sterilization in response to the need for stabilizing the growing population and controlling extensive poverty in the near future [5,6]. Then in early 1980’s the program adopted a new agenda of voluntary acceptance of family planning, and now method acceptance shifted from male to female sterilization [6]. Since then, female sterilization is more common in India than it is anywhere else.

Female sterilization thereafter continued to be the most accessed method of family planning in India. If we have a look on the female sterilization statistics we find that 37% of women in India aged between 15 and 49 are sterilized [7–10]. Most of the sterilization performed is between age 20 and 35years, with half of the women population in India being sterilized by the age of 35 years [6]. About 4.5 million women are being sterilized every year [9]. The National Family Health Survey (NFHS) survey 3 and 4 reveals that one in every three women sterilized reported that they were not informed that it is a permanent method, 68% women i.e. two of every three women reported that they were not informed about the side effects of the method [7–10]. In addition, early marriage and childbearing facilitated the adoption of sterilization at a very young age which is further associated with sterilization regret, particularly for the women without male offspring and child loss [11]. According to the data of United Nations, India alone was responsible for 37 percent of the world’s female sterilization in 2011, in South Asia, India offers the highest percentage of couple adopting this method. A report published by the National Health Mission states that the burden of family planning rests squarely on the shoulders of women in India. The report stated that between 2017–18, 93.1% of the sterilizations performed in India were on women [12]. According to government figures 4.6 million Indian women were sterilized during the year 2011–12 [13]. Data of most recent round of NFHS conducted in 2015–16 offers that about 36 percent of currently married women were sterilization practitioner compared to 37 percent in 2005–06, 34 percent in 1998–99 and 27 percent in 1992–93 [7–10]. On the contrary male sterilization instead of being more cost-effective compared to female sterilization is adopted by very few Indians as a method of family planning [14]. Data on male sterilization shows that it accounts for only 3.31% during NFHS-1 which further declines to 1.69%, 1.05% and 0.34% in NFHS-2,NFHS-3 and NFHS-4 respectively [7–10]. It will not be wrong to say that excessive dependency on of female sterilization debase the promotion and utility of other modern methods.

This excessive dependency on of female sterilization debase the promotion and utility of other modern methods. The announcement made by the Indian government on July 11, 2012, at the London Summit for Family Planning that it has brought about “a paradigm shift” in its approach and will emphasize promotion and provision of contraceptives for birth spacing [13]. Both modern and traditional contraceptive methods also contribute to overall contraceptive use, but their particular share is quite low. Sterilization should be offered only as one of the options among other safe, non-hazardous, non-invasive, long-acting methods of contraception, through an improved basic primary health-care system. Sterilization practice is more common among women belonging to poor communities and those deprived of education [15,16]. Despite the clear distinction in effectiveness between modern and traditional contraceptive methods, a significant number of Indian women still use traditional family planning methods. Several myths about temporary methods of modern contraception restricts many women from accepting short-term reversible methods of contraception and opting for sterilization [6,17]. A number of studies have documented the over emphasis of female sterilization in family welfare programs, poor quality of care and limited choice of methods in both the high and low fertility states [14,15,17]. Inequalities in the provision of family planning and reproductive health information, especially women from poorer communities are disadvantaged in terms of better care and information [11]. There are high unmet needs for modern contraception especially spacing methods, among poor and marginalized women and as a result, they become vulnerable to experiencing poor reproductive outcomes including high rates of unintended and unwanted pregnancies [18]. Ethnicity also plays a source of discrimination for poor women from scheduled tribes(ST), scheduled castes(SC) and other communities [19]. Hounding a poor woman to get sterilized without proper information and leaving her to deal with negative reproductive health consequences cannot be seen as success [13]. The use of female sterilization varies widely across different states in India. For example, in Uttar Pradesh, one of the most economically and socially underdeveloped states in India, only about 17% of women were sterilization users in 2005–2006, compared with 63% in the more developed and urbanized state of Andhra Pradesh [9]. The reason for emphasizing the use of modern contraceptive(reversible) methods is that it acts as a key driver of a diverse set of positive outcomes of women’s health and social well-being [20].

### Aim of the study

Instead of studying the use vs. non use of contraceptives we have focused exclusively on female sterilization use and why its popularity has remained substantial. Considering the above discussion, this study explores the relationship between both past and present context, on women’s contraceptive use in India, in terms of temporary and permanent method over the past two decades. By using data from all four rounds of the NFHS survey, this paper attempts to capture the effect of time as well as the context. In particular, the focus of this paper centers on how this effect is shaped by women’s background and fertility related characteristics. We attempted to explore the following research questions in the present study:

1. What are the trends of different contraceptive methods over the past two decades(1992–2016)?
2. How are women’s socio-demographic and fertility-related characteristics associated with different types of methods?

Following these questions we have also discussed what are the necessary implementation required in the existing family planning programme to promote modern(irreversible) method use among couples that could avert existing dominance female sterilization in future.

Knowledge of female sterilization method and its characteristics delivers a valuable statistic to the implementation of family planning programs and its development in the right direction, which fulfills the need of all the users. Thereafter, this awareness is very important for designing programs encouraging couples to switch from permanent method (i.e. female sterilization) to more reliable and efficient methods of contraception (like reversible modern methods).

### Conceptual framework

**Fig 1:**
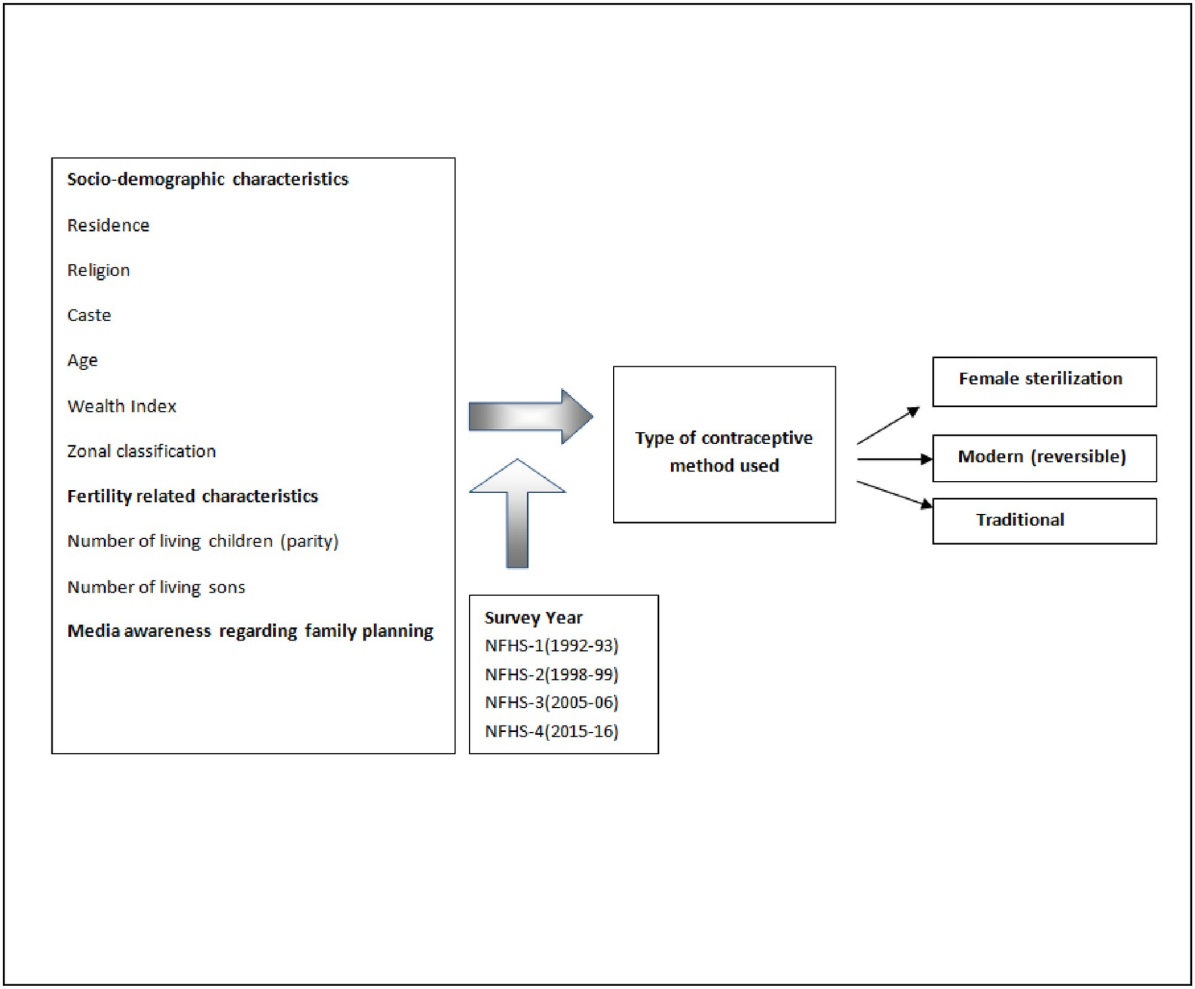
Conceptual framework examining the association between women’s socio-demographic and fertility-related characteristics and type of contraceptive method used.

Fig 1 graphically presents the link between the different factors considered in the study and the choice of contraceptive method. According to the aim of the study we tried to examine the variables that could be relevant to the national family planning program. Various studies have shown a plausible link in socio-demographic factors including residence, ethnicity, religion, wealth status, education level, geographical classification and the pattern of contraceptive use [6,11,21]. Women in India are encouraged for early marriage and childbearing soon thereafter. Once their desired family size is complete they are sterilized [22]. In the present study we have explored how fertility related characteristics are associated with all three contraceptive methods. Exposure to media was considered as it is an effective tool to educate and create awareness among the people.

### Data source and pooling

This study utilizes pooled data from four rounds of 1992–93, 1998–99, 2005–06 and 2015–16 of the NFHS, mainly the women’s file, which gathers information on background characteristics, fertility characteristics, and family planning characteristics of reproductive aged women (15–49) years. NFHS is India’s largest household survey for health planning and policy formulation. The survey was conducted by IIPS, Mumbai funded by ORC Macro and Bill & Melinda Gates Foundation which is the only authentic source which endows a wide range of information on birth histories, maternal and child health conditions at both national and sub national level. The need of pooling arises when we have to analyze larger samples with statistical precision. Here we have used data from the NFHS Surveys 1992–2016 to understand the relationship between socio-demographic and fertility related factors with different types of contraceptive method. Pooling data from four rounds of nationally representative samples is particularly suitable because the samples have been collected using almost similar designs with most of the variables being assigned the similar names and coding. A number of studies [13,14] have adopted the approach of data pooling mainly in the analyses of datasets from the DHS Program.

## Ethics statement

The entire study was based on a large dataset that is publicly available on DHS website (https://dhsprogram.com/data/)conducted by the MoHFW(Ministry of Health and Family Welfare) who has selected International Institute for Population Sciences (IIPS),Mumbai as the nodal agency for conducting NFHS survey in India. All ethical standards have been complied with including informed consent obtained from participants.

### Analytical sample

The analytical sample is confined to 361,402 the currently married and sexually active women aged 15–49 years who responded yes to the question of whether they are currently using any contraceptive method. The 2015–16 NFHS provided a sample of 240553 women, while the remaining 2005–06, 1998–99 and 1992–93 surveys included 48015, 39778 and 33056 women, respectively. The period from 1992–2016 covers more than 20 years which sets a plausible platform for comparison of the trend in the outcome variable i.e. type of contraceptive method used. We have excluded the non-users which will be effective in drawing the influence on the choice between other two methods classified as modern (reversible) and traditional methods, separating the models from the effects of use versus nonuse of method. Before merging the datasets all the variables included in the analysis were harmonized over the four survey years.

### Dependent variable

The outcome variable is the type of contraceptive method used classified into three categories: female sterilization, modern (reversible) and traditional methods. In order to make significant comparison female sterilization is considered the reference category in the analysis. Here the traditional methods comprises rhythm, withdrawal and folk methods while the modern temporary methods comprises the pill, IUD, injectables, condom or any other modern method except male or female sterilization. The counts of male sterilization in the NFHS were is very less (about 1–2%) so was excluded from the present analysis.

### Explanatory variables

On careful examination of the literature review [15,23,24] the explanatory variables are divided into socio-demographic and fertility-related characteristics of women. The socio-demographic variables included are as follows: Age of the respondent which means the age of the women on her last birthday. Here age is taken as a categorical variable and divided into 15–24, 25–34 and 35+ year’s age group. Maternal education corresponds to the highest education the women has attained and is divided into the categories: no education, primary, secondary, and higher education. Wealth index is defined as a composite measure of the socioeconomic status of the women’s household. It is categorized as: poorest, poorer, middle, richer and richest. Residence is defined as the place where women resides which can be an urban or a rural area. The religious affiliation of a women is classified into Hindus, Muslims or others. Here the others category comprises together of Christians, Sikhs, Buddhist/Neo-Buddhist, Jain, Jewish, Parsi/Zoroastrian Donyi polo and others. Women’s caste was classified into three classes namely Scheduled Caste (SC), Scheduled Tribe (ST) and others. Fertility related variables included comprise the following: The number of living children is grouped into 0, 1, 2, 3 and 4+. Similarly, the number of living sons is also divided into 0,1,2,3 and 4+. The other two other variables also included was exposure to family planning awareness and zonal classification. The country was stratified into six zones mainly: North, Central, East, North east, West and South. Since this analysis utilizes pooled data of four surveys we considered time (with year of survey as a proxy) as an independent variable in the regression performed.

### Data analysis

The data of four NFHS surveys were merged into one for performing the pooled analysis. In order to adjust sampling methodologies of the surveys we have used the complex survey module (svyset) to account for primary sampling units different sample weight and sample strata. In order to predict the determinants of modern (reversible) and traditional methods compared to the reference category female sterilization multinomial regression has been performed. The results are reported in terms of relative risk ratios (RRR). The value of RRR>1 means that the independent variable is associated with higher probability of outcome than that of the reference group and RRR < 1 means lower probability of outcome than that of the reference group. During the analysis the survey weights provided by DHS were applied. Before carrying the analysis multicollinearity among explanatory variables was checked by using the technique of variance inflation factor (VIF). The VIF score of 3.16 was obtained which confirmed that there is no serious threat of multicollinearity between the independent variables. Then we have cross-tabulated the frequencies and percentage of each variable. A chi-squared test of association was used to test the statistical significance of the relationship between socio-demographic and fertility related factors with the type of contraceptive method used. The software STATA version 14.0(StataCorp.2015.Stata Statistical Software: Release 14. College Station, TX: StataCorp) was used for data management and analysis. The advantage of modeling the multinomial variables (i.e. sterilization vs. modern vs. traditional) together against two separate binomial logistic models (i.e. sterilization vs. modern; sterilization vs. traditional) is that the previous one allows the random effects in the two contrasts to be correlated. The random error terms are assumed to be independent for both modern and traditional methods. To account for the differential selection probability of each woman we have applied survey weights. The value of alpha is taken to be of 0.05 and 0.01 to test the statistical significance.

## Result

**Fig 2:**
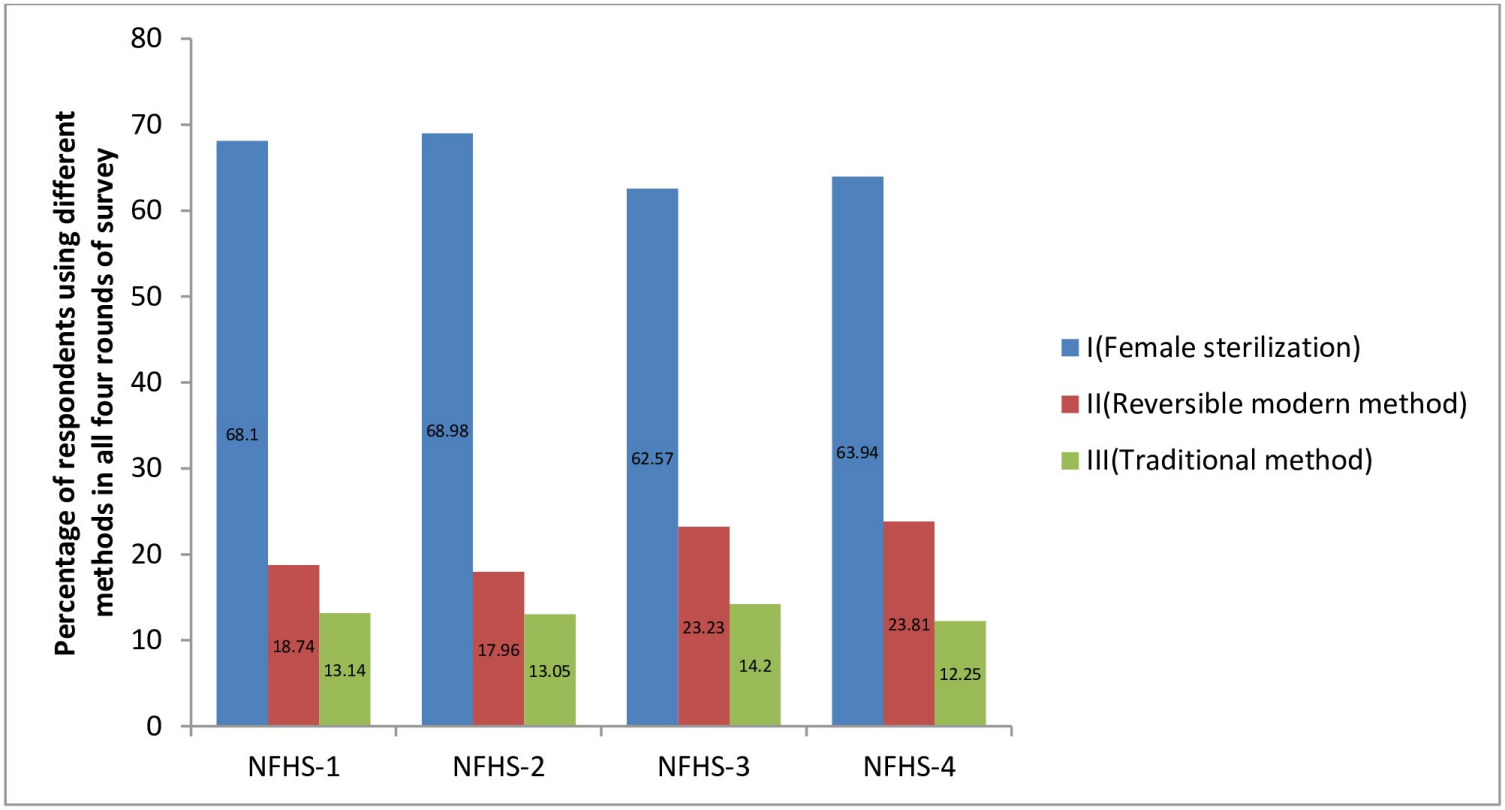
Percentage of contraceptive use according to methods in all four surveys, NFHS I-IV.

Fig 2 illustrates, the changes in different types of contraceptive from 1992 to 2016, with only the female sterilization dominating over the past 20-year period. It is the most widely used contraceptive method in all survey years. The percentage of female using sterilization was 68.1, 68.98, 62.57, and 63.94 in NFHS-1, NFHS-2, NFHS-3 and NFHS-4 respectively. The use of modern methods increased from 18.74 to 23.81 from NFHS-1 to NFHS-4. The traditional method remained almost the same from NFHS-1 to NFHS-4.

Table 1 shows the sample composition of women aged 15–49 years considered in this study. There is a decrement in percentage share of women being sterilized from NFHS-1 to NFHS-4. However, in spite of the decline more than three fifth still use it as a method of birth control which shows that it is the most popular and dominant method of birth control even today in India’s family planning program. A slight increase in modern methods can be noticed during the last decade (2005–2016) whereas the use of traditional methods shows almost no change over time. Majority of the respondent resides in rural areas and belong to Hindu religion. The situation of education has improved over the last two decades. More than two fifth of women were uneducated during NFHS-1, however, this percentage reduced during NFHS-4. Secondary education was the least attained among respondents in NFHS-1 and most in NFHS-4. The proportion of women in the advanced reproductive ages 35 years or above in the sample has increased over the past two decades. Maximum respondents attained parity four or more during NFHS-1 and NFHS-2 but during the last decade (from NFHS-3 to NFHS-4) it reduced to parity three. More than three fifth of women were aware about family planning messages in all the survey years except NFHS-1. The variation in sample composition of wealth index and zonal classification do not follow any trend in the past two decades.

**Table 1:** Sample composition by variable type & survey year: Female age 15–49 years.

Survey Year	NFHS-1		NFHS-2		NFHS-3		NFHS-4	
	N	%	N	%	N	%	N	%
Total	33056	100	39778	100	48015	100	240553	100
**Type of method**								
Female Sterilization	22516	68.1	27440	68.98	30045	62.57	153810	63.94
Modern(reversible)	6195	18.74	7146	17.96	11152	23.23	57265	23.81
Traditional	4345	13.14	5192	1305	6818	14.20	29748	12.25
**Residence**								
Urban	12901	39.03	15137	38.05	23174	48.26	72183	30.01
Rural	20155	60.97	24641	61.95	24841	51.74	168370	69.99
**Religion**								
Hindu	26652	80.63	31866	80.11	38398	79.97	196279	81.59
Muslim	2540	7.68	3718	9.35	4463	9.30	20789	8.64
Others	3894	11.69	4194	10.54	5154	10.73	23485	9.76
**Caste**								
SC	3387	10.25	6308	15.86	8369	17.43	47230	19.63
ST	2711	8.20	3574	8.98	4672	9.73	35258	14.66
Others	26958	81.55	29896	75.16	34974	72.84	158065	65.71
**Maternal education**								
None	14183	44.81	16772	42.16	17197	35.82	85212	35.42
Primary	6794	20.55	7333	18.13	7678	15.99	37577	15.62
Secondary	9170	27.74	11003	27.66	18361	38.24	98164	40.81
Higher & above	2279	6.89	4670	11.74	4779	9.95	19600	8.51
**Women’s age**								
15–24	4052	12.26	4413	11.09	5328	11.10	21615	8.99
25–34	15031	45.47	17472	43.92	20418	42.52	94046	39.10
35+	13973	4.27	17893	44.98	22269	46.38	124892	51.92
**Parity**								
0	439	1.33	409	1.03	669	1.39	3588	1.49
1	2712	8.20	3432	8.63	5606	11.68	29258	12.16
2	8188	24.77	11533	28.99	16861	35.12	94650	39.35
3	9540	28.86	11682	29.37	13057	27.19	63864	26.55
4+	12177	36.84	12722	31.98	11822	24.62	49193	20.45
**Number of living sons**								
0	3355	10.15	3939	9.90	6146	12.80	28920	12.02
1	10721	32.43	14189	35.67	19413	40.43	105444	43.83
2	12285	37.16	14910	37.48	16380	34.11	82559	34.32
3	4766	14.42	4984	12.53	4568	9.51	18347	7.63
4+	1929	5.84	1756	4.41	1508	3.14	5283	2.20
**Heard FP on Radio/TV/newspaper**								
No	14016	42.40	12538	31.52	15081	31.41	84759	35.24
Yes	19040	57.60	27240	68.48	32934	68.59	155794	64.76
**Household member**								
<5	13096	39.62	17806	44.76	26389	54.96	137111	57.0
>5	19960	60.38	21972	55.24	21626	45.04	103442	43.0
**Wealth Index**								
Poorest	3221	9.74	3,956	9.95	4,359	9.08	39272	16.33
Poorer	3881	11.74	5,027	12.64	6,343	13.21	49256	20.48
Middle	5704	17.26	7,453	18.74	8,911	18.56	50937	21.17
Richer	8367	25.31	10129	25.46	12007	25.01	49,522	20.59
Richest	11883	35.95	13213	33.22	16395	34.15	51,566	21.44
**Zonal classification**								
North	8863	26.81	10485	26.36	9190	19.14	57636	23.96
Central	5358	16.21	6726	16.91	9662	20.12	73084	30.38
East	3428	10.37	5174	13.01	5831	12.14	31394	13.05
North East	3064	9.27	3718	9.35	6218	12.95	22363	9.30
West	4828	14.61	5420	13.63	6778	14.12	21584	8.97
South	7515	22.73	8255	20.75	10336	21.53	34492	14.34

### Association between the type of contraceptive method used and socio-demographic and fertility-related characteristics

Table 2 compares the socio-demographic and fertility-related characteristics of current users of female sterilization (**I**), modern method (**II**) and traditional methods (**III**). After analyzing contraceptive prevalence rate according to methods in each survey period, the differentials of contraceptive use by selected background characteristics are computed and results are presented in Table 2. The use of female sterilization is highest in rural area in all surveys, whereas, modern spacing method is highly practiced in urban areas. Female sterilization use is higher in Hindus than Muslims and others; this pattern is consistent for all survey years. By comparing these percentages at different survey time periods we can see that use of modern and traditional methods by Muslims is increasing from 1992–93 to 2015–16. After calculating the diversity according to the caste, the percentage use of modern methods is high in others as compared to SC and ST category in the consecutive surveys. However, SC and ST category women highly rely on the use of female sterilization. Respondent’s education, divided into four parts is related to the level of education of women. Generally, the women who are related to good academic background, have higher knowledge and understanding about different types of family planning methods. It inspires them to use modern methods. Women’s education and modern method choices are positively associated. Highly educated women are more likely to use modern temporary methods compared to those having no education. The percentage use of female sterilization is high among women who are uneducated and this is a constant trend as we move from NFHS-1 to NFHS-4. Respondent’s age, divided in to three parts also show some variation with respect to the use of contraceptive methods. Women whose age group is 35+ years more frequently use permanent method, but women who are between 15 to 24 years prefer modern spacing methods for regulating their fertility. The use of contraceptives is affected by total number of children a woman have at the time of survey as expected. The use of any spacing method either modern or traditional is highest for women who were at parity zero in all surveys, while the pattern of use of permanent method (female sterilization) quietly differs from them, in the earlier surveys (I & II) women adopt sterilization after the parity 4 but the pattern has changed after that, from III round of data we found that women choose this method quite early, at parity 3. The relationship between sterilization choice and number of living sons shows a positive relationship. In India the decision of family size depends upon the sex composition of children mainly on the number of male children. From the tabulation of son composition with the current use of contraceptive method we found that in all the surveys percent of women who have 2 or more sons limiting their fertility by using female sterilization as compared to their counterpart. In absence of any son in the family, modern temporary or traditional methods dominate in all four rounds of survey. Across the four surveys we find a positive impact of the awareness of family planning among the women by means of mass media. Those who were exposed to mass media used modern methods while those not aware preferred the use of female sterilization. Sterilization was equally likely distributed in both sizes of households. Compared with female sterilization, women who use modern methods belong to the highest wealth quantile. This ensures the availability of contraceptives at little or no cost may bridge the gap in modern contraceptive method use between women in the poorest and richest wealth index. Geographical variations were marked as southern zone leads in the female sterilization in all four round of surveys while north east dominates in traditional methods. Region specific estimates offers some decrement in traditional methods from 1992–93 to 2005–06 but again registered some hike in last decade.

**Table 2:** Percentage distribution of contraceptive methods use by the background characteristics for NFHS I-IV.

Method mix conditional on use(%)												
Variables	NFHS-1			NFHS-2			NFHS-3			NFHS-4		
	I	II	Ill	I	II	III	I	II	III	I	II	Ill
**Residence**												
Urban	56.79	28.97	14.24	58.85	26.52	14.63	56.36	29.61	14.02	57.29	30.53	12.18
Rural	75.37	12.19	12.44	75.21	12.71	12.09	68.37	17.27	14.36	66.79	20.92	12.92
**Religion**												
Hindu	70.93	17.20	11.87	72.13	15.69	13.90	12.19	20.93	13.90	67.68	20.61	11.71
Muslim	53.07	24.69	22.24	55.08	27.30	17.62	49.83	33.86	16.31	42.43	38.57	19.00
Others	58.57	25.49	15.94	57.42	26.99	14.63	54.23	31.14	14.63	51.74	37.42	10.84
**Caste**												
SC	77.77	12.11	10.13	77.33	11.51	11.16	68.72	18.02	13.20	68.22	20.11	11.67
ST	76.50	13.46	10.03	70.76	17.29	11.95	63.48	22.71	13.81	62.43	26.05	11.52
Others	66.06	20.11	13.84	67.01	19.41	13.58	60.98	24.53	14.49	63.00	24.41	12.59
**Maternal Education**												
None	81.72	8.92	9.36	81.89	8.25	9.86	76.78	11.55	11.68	76.96	12.41	10.63
Primary	74.58	13.59	11.83	76.03	13.42	10.56	72.49	15.99	11.51	70.63	18.71	10.66
Secondary	52.78	29.41	17.81	60.05	24.49	15.46	54.41	29.45	16.13	56.17	30.35	13.48
Higher and above	22.11	54.98	22.90	32.61	44.60	22.78	26.89	52.94	20.17	33.43	50.32	16.24
**Women’s age**												
15–24	38.03	38.60	23.37	40.11	37.64	22.25	30.69	45.74	23.57	24.86	51.87	23.27
25–34	66.32	21.96	11.72	65.54	21.81	12.65	56.86	28.95	14.19	55.39	31.38	13.22
35+	78.77	9.52	11.71	79.47	9.36	11.17	75.44	12.59	11.97	77.14	13.24	9.62
**Parity**												
0	5.24	44.87	49.89	7.09	39.61	53.30	3.14	52.02	44.84	6.33	64.02	29.65
1	13.94	51.95	34.11	14.31	50.70	34.99	12.18	55.67	32.14	18.42	56.48	25.10
2	55.25	28.11	16.63	62.79	23.72	13.49	61.59	25.91	12.50	66.67	23.19	10.14
3	79.09	12.46	8.45	80.62	11.05	8.33	77.70	13.46	8.84	76.65	14.75	8.60
4+	82.50	9.02	8.48	80.66	9.57	9.78	74.54	13.16	12.30	73.46	14.38	12.16
**No. of living sons**												
0	29.60	39.40	31.00	33.84	37.52	28.64	31.79	41.39	26.81	31.39	46.88	21.73
1	56.11	27.25	16.64	59.43	24.68	15.89	56.11	28.61	15.27	59.45	27.55	13.00
2	80.45	11.75	7.80	81.29	10.62	8.08	77.06	13.86	9.08	77.94	13.87	8.19
3	85.42	7.26	7.32	84.15	7.76	8.09	77.96	11.58	11.58	77.36	12.69	9.94
4+	80.51	8.40	11.09	77.45	11.10	11.45	67.24	16.84	15.92	66.36	16.64	17.00
**FP Awareness**												
No	77.20	10.12	12.67	80.79	8.96	10.25	73.87	13.45	12.68	69.01	17.74	13.25
Yes	61.42	25.08	13.49	63.55	22.11	14.34	57.40	27.70	14.90	61.18	27.10	11.71
**Household member**												
<5	65.57	19.72	14.71	68.62	17.94	13.44	63.95	22.19	13.85	66.07	22.62	11.31
5+	69.8	18.10	12.11	69.27	17.99	12.74	60.89	24.48	14.62	61.11	25.38	13.51
**Wealth Index**												
Poorest	79.88	6.09	14.03	80.06	6.62	13.32	72.84	10.16	17.00	70.37	15.31	14.32
Poorer	78.25	7.55	14.20	80.01	8.51	11.48	72.39	12.97	14.63	66.55	20.10	13.35
Middle	80.40	8.84	10.76	79.94	10.33	9.73	70.54	15.95	13.51	67.16	21.37	11.47
Richer	73.28	15.67	11.06	71.88	16.01	12.10	65.16	21.86	12.98	64.43	24.51	11.06
Richest	52.08	32.74	15.17	53.07	30.76	16.17	49.82	35.62	14.56	52.90	35.54	11.56
**Zones**												
North	58.41	30.11	11.47	61.76	25.34	12.89	56.92	34.22	8.86	59.16	30.81	10.03
Central	73.57	20.74	5.69	74.20	14.91	10.88	59.15	23.78	17.07	63.78	20.46	15.76
East	63.19	11.46	25.35	55.12	19.29	25.59	52.62	23.15	24.23	56.01	26.80	17.18
North East	42.62	20.79	36.59	46.69	27.54	25.77	33.98	34.19	31.83	27.59	48.50	23.91
West	77.38	13.88	8.74	76.20	15.96	7.84	70.66	20.88	8.47	79.52	16.38	4.10
South	82.36	9.51	8.13	87.89	7.26	4.86	88.32	7.91	3.76	93.30	5.10	1.60

## Regression results

In order to demonstrate whether the differences offered by the covariates used in the study were statistically significant or not and how their movement from one socio-demographic group to another affected the demographic profile of women at sterilization in relatively heterogeneous population of India, we had used multinomial regression. Table 3 reports adjusted relative risk ratios(RRR) based on the pooled data. Female sterilization being considered as a reference category, compared with modern and traditional methods. Table 3 reports that women in survey year 2006(NFHS-3) and 2016(NFHS-4) have higher probability of using modern contraceptive method compared with women in 1992(NFHS-1). This supports the fact that, over time, modern method use has been increasing in the India. The relative risk became significant for the survey year 2016 where traditional method use had 23% lower risk as compared to sterilization. This justified that female sterilization was still dominant when compared to traditional methods at the present time. The RRR showed that urban women were relatively less likely to prefer sterilization as compared to temporary (reversible modern and traditional) methods. Considering the religious affiliations for both modern and traditional method use the relative risk was greater than one, indicating that the use of modern methods by Muslim women was more than four times and traditional methods more than three times (RRR = 4.52,95% C.I: 4.18–4.88 for II & RRR = 3.11, 95% C.I: 2.86–3.40 for III) as compared to Hindus. Similarly, other category women(RRR = 1.55,95% C.I: 1.44–1.67 for II & RRR = 1.48, 95% C.I:1.43–1.63 for III)were more likely to use modern and traditional methods over female sterilization as compared to the Hindus. However, the caste offered significant differences only for modern methods versus sterilization suggesting that Schedule tribes(STs) relied more on female sterilization when compared to the Schedule castes(SCs) having relative risk less than one(RRR = 0.77, 95%C.I:0.71–0.85). Education appears to exert expected influence on the use of contraceptive method. As we move from no education to higher education category we found a significant increase in the relative risk ratios. Educated women were highly more probable to use temporary methods over sterilization. For both modern and traditional methods, the relative risk ratios of choosing either method over female sterilization methods by age had values below one, suggesting that younger women were less probable to use female sterilization method over temporary methods compared with women aged 35+, who were approaching to the end of their reproductive years. Table 3 highlights a positive relationship between parity and use of female sterilization which meant that as number of living children increased women move towards sterilization. Higher parity women were at a greater risk of using permanent method as compared to women at low parity. The relative risk for using modern contraceptive and traditional method relative to female sterilization reduced by a factor of 0.55 for women with one son. Likewise, it reduced by a factor of 0.29 for women with two sons and 0.49 for women with more than four sons. This showed that women with two or more sons had less probability of using modern or traditional methods, and higher probability for permanent method. Exposure to family planning awareness appears to be significantly associated with the types of contraceptive method used. The relative risk for modern and traditional method relative to female sterilization increased by a factor 1.30 and 1.04 respectively. This meant that women with exposure to family planning awareness had higher likelihood of using modern and traditional methods as compared to sterilization. The chance to use modern contraceptives increased with the increase in wealth index. The relative risk ratio for using modern contraceptives relative to female sterilization increased by a factor of 1.63 for women belonging to wealthiest index. However, the result was not significant for traditional methods. The expected risk of using modern contraceptive & traditional method relative to use of sterilization increased by a factor of 1.24 and 1.22 for women who had large family size compared with women who had small family size. The disparity among zonal classification was obvious with previous studies [25,26]. The relative risk ratio of using modern and traditional method relative to sterilization showed that female sterilization leads in the southern region of India. However, relative risk of using modern and traditional method in north east region increased by the factor of 2.46 and 7.5 respectively.

**Table 3:** RRR showing influence on use of contraceptive with sterilization method as base outcome(pooled data 1992–2016).

Background Characteristics	Modern(reversible) vs. Sterilization	Traditional vs. Sterilization
	Relative risk ratio (95%C.I.)	Relative risk ratio (95%C.I.)
**Survey Year**		
1993	**Reference**	**Reference**
1999	0.874*(0.806–0.947)	0.969 (0.878 1.069)
2006	1.205**(1.113–1.304)	1.207 **(1.100–1.325)
2016	1.152**(1.079–1.231)	0.771**(0.710–0.837)
**Religion**		
Hindu	**Reference**	**Reference**
Muslim	4.521** (4.185–4.883)	3.114** (2.859–3.391)
Others	1.553 **(1.444–1.671)	1.478**(1.342–1.627)
**Caste**		
SC	**Reference**	**Reference**
ST	0.773**(0.708–0.845)	0.588(0.528–0.655)
Others	0.994(0.940–1.051)	0.967(0.905–1.032)
**Maternal Education**		
None	**Reference**	**Reference**
Primary	1.431**(1.349–1.518)	1.104**(1.039–1.173)
Secondary	2.494**(2.360–2.635)	1.812**(1.710–1.920)
Higher and above	6.642**(6.156–7.167)	3.738**(3.408–4.099)
**Women’s age**		
15–24	**Reference**	**Reference**
25–34	0.439**(0.415–0.463)	0.538**(0.506–0.572)
35+	0.164**(0.154–0.175)	0.415**(0.387–0.446)
**Parity**		
0	**Reference**	**Reference**
1	0.930*(0.759–1.140)	0.541**(0.441–0.664)
2	0.154**(0.126–0.190)	0.073**(0.060–0.090)
3	0.099**(0.804–0.122)	0.048**(0.039–0.060)
4+	0.123**(0.099–0.151)	0.064**(0.051–0.079)
**No. of living sons**		
0	**Reference**	**Reference**
1	0.553*(0.523–0.585)	0.503*(0.472–0.536)
2	0.287*(0.269–0.306)	0.270*(0.251–0.290)
3	0.290*(0.264–0.319)	0.302*(0.275–0.331)
4+	0.491(0.432–0.559)	0.417(0.369–0.473)
**FP Awareness**		
No	**Reference**	**Reference**
Yes	1.295**(1.237–1.354)	1.044(0.992–1.100)
**Household member**		
<5	**Reference**	**Reference**
5+	1.239**(1.191–1.288)	1.225**(1.171–1.281)
**Wealth Index**		
Poorest	**Reference**	**Reference**
Poorer	1.026(0.948–1.110)	0.836**(0.778–0.899)
Middle	0.990(0.914–1.072)	0.738**(.681–0.799)
Richer	1.179**(1.085–1.282)	0.764**(0.700–0.834)
Richest	1.629**(1.485–1.787)	0.941(0.853–1.039)
**Zones**		
North	**Reference**	**Reference**
Central	0.874**(0.821–0.930)	1.629**(1.508–1.760)
East	0.768**(0.710–0.830)	1.891**(1.732–2.064)
North East	2.462**(2.196–2.761)	7.505**(6.656–8.463)
West	0.268**(0.248–0.289)	0.269**(0.242–0.299)
South	0.067**(0.062–0.072)	0.117**(0.106–0.129)
**Residence**		
Rural	**Reference**	**Reference**
Urban	1.382**(1.305–1.465)	1.073*(1.001–1.151)

*p < 0.05.

**p < 0.01.

RRR: Relative Risk Ratio.

## Discussion and conclusion

Undoubtedly, the use of contraceptive pattern in India was at a different paradigm twenty years back. However, the result of this study clearly demonstrates the use of female sterilization remained substantial over past two decades. A closer examination over different types of method use, however, may suggest a slight decreasing percentage of traditional methods and increasing percentage of modern methods over time from NFHS-1 to NFHS-4. This shows that women in 1990’s were less likely to prefer modern methods as compared to women in 2016. This result is supported by many other studies [26,27]. This result highlights the modest success of family planning programs in promoting the use of modern methods. There was a high degree of consistency in the demographic information obtained from the four surveys. As per the data of India’s Ministry of Health and family welfare about 2 million India couples adopt female sterilization as a method of birth control [28]. The findings of the study suggest that sterilization is at higher risk among women with lower wealth quintile, uneducated or less educated, having higher parity and who are less exposed to media.

Religion wise comparison revealed that Muslim women dominates in using temporary methods while Hindu women prefers female sterilization. The reason for low prevalence of sterilization and high utilization of temporary methods by Muslim women can be explained by the religious doctrine of Islam which does not favors permanent methods of family planning [29–31]. The longstanding heterogeneity of population of India associated with religion and the caste system is still relevant in contraceptive choices [15–17,23,32,33].

Educational status of women offers a positive engagement with different method choices. A positive effect of women’s education can be noticed on modern contraceptive method use. As evident from the regression analysis, highly educated women are more likely to use modern methods. However, the result also confirmed the high level use of traditional methods among educated women. This result was surprising although there are many other studies support this fact [17,23]. A study also showed that one of the greatest factors supporting traditional contraception use among more educated and wealthier women was not the perceived ineffectiveness of modern methods but their wholesome side effects [33].

The prevalence of sterilization rises with the increase in age and becomes the maximum in age group 35-45years. Considering the age of women, clearly younger age group women had adopted permanent method in the decade of 1990’s as compared to women in survey of 2015–16. This can be attributed to the fact of declining trend of age at marriage before 18 years in the past 10 years [25].

Parity turned out to be a strong determinant for women choosing sterilization. The result of the analysis explores the ways in which decision making on female sterilization is affected by parity of a women as well as number of sons. At lower parity temporary methods dominate while as parity increases women switch to female sterilization. Difference in the average parity among the groups offered a decreasing trend which concludes that there is a decline in parity among the sterilized women from NFHS-1 to NFHS-4. At higher parity levels and those women with two or more surviving sons were much more inclined towards female sterilization. So, we can conclude that sterilization is regulated by parity of a couple. Another important result is the increasing percentage of modern method use by women at lower parity as compared to traditional methods in the last round of survey i.e. NFHS-4 (2015–16). This means that women have started to rely on modern methods in the last few years as compared to traditional method which can be considered as a modest success of family planning program in promoting modern methods. The present study also shows that relative risk of using sterilization increases with the increase in number of sons in all four rounds of survey. This result can be attributed to the fact that women in India start using sterilization when they have fulfilled their goal of ideal number of sons. This conclusion is supported by many other studies [24,34–37].

A positive impact of awareness through mass media can be noticed similar to many other studies [25,27,33]. Women who were exposed to the mass media prefer temporary methods over permanent. This finding suggests that we can educate the women through mass media awareness programs about the effectiveness of temporary methods and motivate them for the adoption of modern methods.

Reliance over female sterilization was observed in almost all parts of the country but the prevalence was not uniform in all the zones. The exceptionally high acceptance of sterilization in the southern region could be correlated with high demand for limiting fertility and better network of family planning services. In contrast, the physical access to reproductive health services is generally poor in larger high fertility states located in northern and central regions. More than 80% of women in southern region of the country rely on female sterilization from the past two decades. The other reason for this high prevalence is attributed by factors such as temporary alternatives are still considered a taboo, lack of awareness, incentives provided for sterilization which will not be provided if they use any other method and attitude of males for not taking equal responsibility of contraception [38,39]. Zonal classification generally displayed distinctions consistent with the previous studies stating that sterilization in India is geographically concentrated in southern region [4,9]. Public sector is the backbone of providing family planning services in India which lacks in terms of proper training, efficient manpower, poor infrastructure and weak coordination in all its aspects [40]. The poor quality of these services and exclusive focus limited to permanent methods prevent people to adopt modern methods of contraceptives [40–42]. The poor and uneducated population of the country totally depend on these services. Clearly, the foregoing analyses confirm the persistent dominance of sterilization use amongst the poorer strata of the Indian society highlights the weakness of the national family planning program in promoting wider method choices. So, there is a need to strengthen these services in the right direction promoting the effective modern methods of family planning instead of making them rely only on permanent methods.

Many new initiatives by the government has been launched in this direction like 360 degree media campaign, home delivery of contraceptives by ASHA Workers, launch of population stabilization mission by the name Pariwar Vikas Mission and spreading awareness among the masses regarding reversible methods of contraceptives [43]. The Indian government announced that its new strategy focuses on “making contraceptives available at the doorstep through community health workers,” especially in those public health facilities that have large numbers of women coming to give birth [13].

Our study shows that more than two-third of currently married women rely on sterilization as a method of birth control over two decades. With the present approach of family planning program in India to focus only on addressing the unmet need, an understanding of the factors promoting female sterilization use is imperative. If sterilization remains in such high demand women will continue seeing it as their best option. An example of Bangladesh can be considered where female sterilization declined drastically with promotion of modern methods in their family planning programme [44]. A shift from permanent to temporary contraceptive(reversible) methods is necessary as it acts as a key driver of a diverse set of positive outcomes of women’s health and social well-being [20]. This study concludes that ongoing family planning programme in India have created an coercive environment for female sterilization over the past two decades to achieve their numerical targets. So, there is a need to implement the existing family planning programs by promoting wider choices of contraceptives among couples and encouraging couples to switch from permanent method (i.e. female sterilization) to more reliable and efficient methods of contraception (like reversible modern methods).

### Strength and limitations

A particular strength of this study is that we conducted this analysis using nationally representative data from four rounds of NFHS which enables us to understand the transition in demographic profile of sterilized women over the past two decades (i.e. from 1992 to 2016) and their association with relevant socio-demographic and fertility related indicators. This allowed us to investigate and observe trends and relationships that would help across programmatic and development contexts. We cannot deny from the limitations we encountered in this study. There are certain variables that could have been used to enrich this study like variables governing women autonomy and decision making power but due to fewer observations we have skipped them. The non-users of the contraceptives were not considered as it was beyond the scope of this study. The chances of under reporting cannot be denied as contraceptive is treated as a very sensitive and stigmatized subject in country like India, so respondents may refuse to disclose their responses. NFHS fails to provide data on household income and expenditure so we were forced to use a proxy variable wealth index based on assets.
